# Investigating Relationships Between Alcohol and Cannabis Use in an Online Survey of Cannabis Users: A Focus on Cannabinoid Content and Cannabis for Medical Purposes

**DOI:** 10.3389/fpsyt.2020.613243

**Published:** 2020-12-21

**Authors:** Hollis C. Karoly, Raeghan L. Mueller, Chrysta C. Andrade, Kent E. Hutchison

**Affiliations:** ^1^Institute for Cognitive Science, University of Colorado Boulder, Boulder, CO, United States; ^2^Department of Psychology, Colorado State University, Fort Collins, CO, United States; ^3^Department of Psychology and Neuroscience, University of Colorado Boulder, Boulder, CO, United States; ^4^Department of Psychiatry, University of Colorado Anschutz Medical Campus, Aurora, CO, United States

**Keywords:** alcohol, cannabis, tetrahydrocannabinol (THC), cannabidiol (CBD), polysubstance use

## Abstract

Cannabis is commonly used among people who drink alcohol, but findings are mixed regarding the direction of this relationship. The type of cannabis used [high-cannabidiol (CBD) vs. high-delta-9tetrahydrocannabinol (THC)] and motives for use (i.e., whether cannabis is used to treat a medical condition) may influence the relationship between cannabis and drinking. Specifically, CBD has shown preclinical promise in reducing alcohol consumption, and medical cannabis users report using cannabis to reduce drinking. This study leverages survey data from cannabis users who drink alcohol (*N* = 533). Respondents were categorized as using cannabis to treat (CTT) a medical condition or as individuals whose cannabis use is not intended to treat (NCTT) a medical condition and grouped based on the THC/CBD ratio of the flower or edible cannabis they typically use (e.g., “High-THC/CBD,” “Medium-THC/CBD” and “Low-THC/CBD”). The CTT group (*n* = 412) reported drinking significantly less frequently than the NCTT group (*n* = 121). Cannabinoid content of flower cannabis was associated with alcohol consumed on cannabis-use days, such that individuals in the High-THC/CBD group drink more on cannabis-use days compared to the Medium-THC/CBD group. Cannabinoid content of edible cannabis was associated with drinks per drinking occasion, such that the High-THC/CBD group consumed the most drinks and the Low-THC/CBD group consumed the fewest. For both edible and flower groupings, higher-THC/CBD cannabis was associated with more frequent co-use than lower-THC/CBD cannabis. Results suggest that whether someone uses cannabis to treat a medical condition may impact their drinking frequency, and the cannabinoid content in flower and edible cannabis impacts alcohol consumption.

## Introduction

Amidst the changing legal landscape surrounding cannabis in the United States, cannabis and alcohol co-use is becoming increasingly common (1). However, insufficient research exists to clarify the effects of cannabis use on alcohol consumption patterns. Studies in this area have been conflicting, with some suggesting that cannabis use is associated with increased drinking (i.e., “complementarity”) and others suggesting that cannabis decreases alcohol consumption (i.e., “substitution”) (2, 3). Motives for use (e.g., using to treat a medical condition) and cannabinoid content [e.g., high-cannabidiol (CBD) vs. high-delta-9tetrahydrocannabinol (THC)] may impact the association between cannabis and alcohol use.

A recent systematic review on alcohol and cannabis substitution and complementarity, which included 64 articles spanning human and animal studies, found that 30 studies supported substitution, 17 suggested complementarity, 14 supported neither and 4 supported both (4). One notable finding from this review was that all studies conducted on medical cannabis patients supported substitution. Specifically, one U.S.-based study found that 40% of medical users report using cannabis to decrease alcohol intake (5). Another study conducted across three U.S. dispensaries found that participants reported a 42% reduction in alcohol consumption after they started using medical cannabis (6). Three Canadian studies reported that participants substitute medical cannabis for alcohol at a rate of 25–52% (7–9). Further, alcohol consumption has decreased significantly in states with legalized medical cannabis (10), and medical users have been shown to drink less and have fewer alcohol-related problems than recreational users (11). Conversely, one study using data from the National Survey on Drug Use and Health found that individuals in states that had implemented medical cannabis legalization were more likely to engage in binge drinking (12).

No prior studies have explored how cannabinoid content influences the relationship between cannabis and alcohol. A recent review of the existing evidence suggested that CBD may mitigate symptoms of alcohol use disorders (AUD) (13). Although little work has been done in this area among humans, preclinical literature shows that CBD decreases drinking motivation and consumption and reduces the reinforcing properties of alcohol in mice (14–16), and decreases cue- and stress-induced alcohol-seeking, reinstatement, anxiety, and high impulsivity in rats (17). The preclinical literature on the impact of THC on alcohol consumption is inconsistent. THC *decreases* alcohol intake in rats (18) and inhibits locomotor sensitization (a rodent marker of dependence) induced by ethanol (19), suggesting that THC is associated with decreased alcohol consumption. Conversely, CB_1_ knockout mice (i.e., mice lacking in the cannabinoid receptor to which THC binds) show reduced alcohol intake (20), and alcohol intake is also reduced by CB_1_ antagonists (21), suggesting that activation of CB_1_ by THC may be associated with greater alcohol intake.

No published human laboratory studies to our knowledge have used alcohol and cannabis co-administration procedures to explore the impact of acute cannabis use on alcohol consumption within a given co-using session. However, as reviewed in Yurasek et al. (22), national survey data suggest that simultaneous alcohol and cannabis co-use is associated with increased quantity and frequency of drinking (1) and that individuals who report higher levels of cannabis use generally report greater alcohol consumption compared to those who use less cannabis (23). Similarly, college students who drink heavily are more likely to have used cannabis in the past year compared to those who drink less (24) and those who use cannabis are more likely to drink alcohol, binge-drink and experience adverse alcohol-related outcomes (25).

The present study leverages a convenience sample of online survey data to compare alcohol use patterns across cannabis users who were identified as using cannabis to treat a medical condition (CTT) and individuals who report that their cannabis use is not intended to treat a medical condition (NCTT). We also compare outcomes across individuals who report different THC/CBD ratios in their typical flower and edible cannabis. Cannabinoid content is an important and novel variable that is not typically included in survey research on alcohol and cannabis use. We hypothesize that the CTT group will report (1) lower drinking frequency, (2) fewer drinks per drinking occasion (DPDO), (3) lower alcohol and cannabis co-use frequency, and (4) indicate that they drink less alcohol on days when they use cannabis compared to NCTT users. We further hypothesize that those who use cannabis with Low-THC/CBD ratio will report (1) lower drinking frequency, (2) fewer DPDO, (3) lower co-use frequency, and (4) indicate that they drink less alcohol on days when they use cannabis compared to individuals who consume cannabis containing a Medium- or High-THC/CBD ratio. We also hypothesize that those using High-THC/CBD cannabis will report higher scores on all outcome measures compared to those using Medium-THC/CBD cannabis.

## Methods

### Procedures

The study was approved by our University's Institutional Review Board. Our voluntary, anonymous survey was hosted on Qualtrics.com and distributed on social media from May 2017 to January 2020. The social media advertisement targeted individuals aged 21 and older living in states with legal access to medical and recreational cannabis and who “liked” cannabis-related pages (e.g., on Facebook, Instagram, Reddit, Tumblr). The advertisement was also posted at local medical and recreational cannabis clinics and advertised on the radio, online news sources and our university website. The advertisement asked prospective respondents if they are “interested in contributing to research regarding cannabis and health.”

Of the respondents included in this sample (*N* = 533), 232 reported that they saw the advertisement on social media, 158 saw it at a cannabis clinic, 3 heard about it on the radio, 9 saw it on the university webpage, 1 saw it in an online newspaper, 87 did not disclose where they saw it and 43 reported hearing about it in some other way, such as word of mouth. Anyone 21 years of age or older was allowed to take the survey. Interested individuals clicked on the Qualtrics link that directed them to the informed consent page. This page explained the purpose of the survey and participation was clearly stated as voluntary, with the option to withdraw at any time. Respondents who provided consent were re-directed to the survey hosted through Qualtrics. The survey took ~15 min to complete. Participants were not compensated for participation.

### Measures

Participants were queried on demographics, substance use and health. They were asked how often they used various cannabis products (e.g., flower cannabis, edible cannabis) on a 13-point scale ranging from “*Never*” to “*Daily use*.” Note that some individuals took the survey despite not being cannabis users (i.e., indicating “never” for all forms of cannabis use). They were excluded from all analyses. Participants were asked to indicate the potency of THC or CBD is in the product(s) they typically use. Estimates for cannabinoid concentrations were provided as percent THC/CBD (potency) for flower and THC/CBD milligrams for edibles. Cannabis products purchased from dispensaries are required to have their THC and CBD content listed on the packaging, so it is reasonable to expect that individuals taking the survey would know their product's content. All subjects provided estimates of the THC and CBD content of their typical cannabis.

Respondents were also asked whether they drank alcohol (yes/no) and if “yes,” they were asked how often they drink on a 7-point scale ranging from “*Less than once a month*” to “*Daily*.” They were asked to indicate how many drinks they consume on average when they drink, with standard equivalents provided for beer (12 oz), wine (5 oz), and hard liquor (1.5 oz). Individuals were asked to indicate on a 7-point scale, “*How often do you use cannabis and drink alcohol at the same time? (Using one while feeling the effect from the other)*” with responses ranging from “*Never*” to “*Every day*.” Respondents were asked to indicate on a 5-point Likert scale, “*On the days when you use cannabis, do you usually drink more alcohol than usual, less alcohol than usual, or about the same amount?*” with responses ranging from “*Much less alcohol*” to “*Much more alcohol*.”

Participants were asked whether they have been diagnosed with or experience medical issues commonly reported by medical cannabis patients. They were asked to use a nominal yes/no scale to indicate whether they experience any of the following conditions: chronic pain, migraines, anxiety or depression, cancer, post-traumatic stress disorder (PTSD), a sleep disorder (e.g., insomnia, sleep apnea) or any “other” condition (they were provided a text field to state the condition). Chronic pain, migraines, anxiety, depression, cancer, post-traumatic stress disorder (PTSD) and sleep disorder were included as specific questions in the survey due to substantial evidence that they are common conditions for which people seek out medical cannabis (26, 27). Participants were then asked whether they use cannabis to treat each condition(s) that they endorsed experiencing (including anything they listed in the “other” category).

### Creation of Variables for Analysis

Survey participants were cannabis users who were categorized into groups based on whether they (1) use cannabis to treat a medical condition (CTT) or whether their cannabis use is not intended to treat a medical condition (NCTT), and (2) according to the average THC/CBD ratio in the edible and flower cannabis that they typically use. Participants were classified as CTT (*n* = 412) if they reported using cannabis to alleviate symptoms of any of the medical conditions queried in the survey or for any “other” medical reason; otherwise, they were classified as NCTT (*n* = 121).

To classify participants according to the average THC/CBD ratio in the cannabis flower that they reported smoking most often, we used responses to “*How much THC is in the cannabis flower that you smoke most often?*” and “*How much CBD is in the cannabis flower that you smoke most often?*” If they used a ratio of 10:1 THC/CBD or higher, they were classified in the High-THC/CBD flower group (*n* = 182); if they used a ratio of 1:1 THC/CBD or less, they were classified in the Low-THC/CBD flower group (*n* = 113) and if they used any ratio of THC/CBD above 1:1 and below 10:1, they were classified in the Medium-THC/CBD flower group (*n* = 195).

Similar groupings were created based on participants' self-reported content of the edible cannabis they typically use. Responses to “*On average, how many milligrams (mg) of THC do you consume at one time when using an edible*” and “*On average, how many milligrams (mg) of CBD do you consume at one time when using an edible*” were used to create the same categories for edible cannabis use. If participants reported using a ratio of 10:1 THC/CBD or higher, they were classified in the High-THC/CBD edible group (*n* = 99); if they used a ratio of 1:1 THC/CBD or less, they were classified in the Low-THC/CBD edible group (*n* = 143); and if they used any ratio of THC/CBD above 1:1 and below 10:1, they were classified in the Medium-THC/CBD edible group (*n* = 174). If individuals reported using “0” THC and >0 CBD, they were classified in the Low-THC/CBD group, and if they reported “0” CBD and >0 THC, they were classified in the High-THC/CBD group. Note that commercial CBD products are typically extracted from whole hemp plants and include traces of other cannabinoids, including THC (28), and even cannabis plants bred to be high in CBD contain trace amounts of THC (29). For this reason, considering individuals who used some CBD and “0” THC in the Low-THC/CBD group is appropriate, as they likely are consuming very low levels of THC in their high-CBD products.

Note that some individuals reported only flower (no edible) use; they were only included in the analyses using the flower groupings and comparing CTT to NCTT groups. Some individuals reported only edible (no flower) use; they were included only in analyses using the edible groupings and comparing CTT to NCTT. Individuals could be in different cannabinoid groups for flower and edible if they reported using different THC/CBD ratios in their flower and edible products. For example, if someone reported typically using a high THC, low CBD edible, they would be in the High-THC/CBD group for the analyses using the edible-based groupings. However, if they also used a low THC, high CBD flower product, they would be included in the Low-THC/CBD group for analyses using the flower-based groupings.

### Data Analytic Strategy

Data were analyzed using SPSS (Version 27). To analyze demographic differences between CTT and NCTT users, independent samples *t*-tests were conducted on continuous variables (e.g., age), and chi-squared tests were conducted on categorical variables (education, gender, and employment status) (Table 1). To analyze demographic differences across the cannabinoid groupings, ANOVA was performed on age and chi-square tests were conducted on categorical variables. Gender differed across the CTT and NCTT groups (Chi square = 10.97, *p* = 0.001), with a larger percentage of males in the NCTT group. Age and employment were different across the flower groupings (*p* < 0.001), with the Low-THC/CBD group being the oldest and containing a higher percentage of unemployed, disabled or retired individuals (Chi Square = 16.43, *p* = 0.037). Age was different across the edible groups (*p* < 0.001), with the Low-THC/ CBD group being the oldest. Thus, gender was included as a covariate in CTT vs. NCTT analyses, age and employment were included in analyses using the flower groupings, and age was included in analyses using the edible groupings. Six participants did not provide their gender, five did not provide their age and four did not provide employment information.

**Table 1:** Demographic characteristics for individuals who use cannabis to treat a medical condition (CTT) and individuals whose cannabis use is not intended to treat a medical condition (NCTT).

**Characteristic [Mean (SD)]**	**Overall (*N* = 533)**	**CTT (*n* = 412)**	**NCTT (*n* = 121)**	***p*-Value**
**Demographics**				
Age	34.9 (14.3)	35.07 (14.0)	34.1 (15.6)	0.530
**Gender (% female)**	**43.7%**	**47.3%**	**29.8%**	**0.001**
Race (% white)	76.5%	78.7%	74.1%	0.294
Education (% bachelors or higher)	39.8%	39.8%	38.8%	0.951
Employment (% full time employed)	58.0%	56.6%	61.7%	0.359

*p-values associated with chi-square tests for categorical variables and t-tests for age. For race, tests were run across groups comparing white individuals vs. all other racial identifications, for education they were run comparing bachelors or higher vs. less than bachelors and for employment they were run comparing full time employed vs. all other employment statuses. Note that not all subjects answered every question so group ns for each demographic variable may be less than total n for that group. Significant group differences between CTT and NCTT are denoted by bold text*.

We ran Ordinary Least Squares (OLS) regression models in which hypotheses were tested using two orthogonal contrast codes to examine group differences in drinking frequency, DPDO, co-use frequency, and response to the question: “*On the days when you use cannabis, do you usually drink more alcohol than usual, less alcohol than usual, or about the same amount?*” To test the hypothesis that the low-THC/CBD group will drink less than the other two groups, the low-THC/CBD group was coded as “−2,” and the Medium- and High-THC/CBD groups were both coded as “1” (Contrast 1). To test the hypothesis that the High-THC/CBD group will drink more than the Medium-THC/CBD group, the Low-THC/CBD group was coded as “0,” the High-THC/CBD group was coded as “1” and the Medium-THC/CBD group was coded as “−1” (Contrast 2). In each model, the outcome of interest (e.g., “DPDO”) was regressed on both contrast codes and relevant covariates^1^.

## Results

### Participant Characteristics

A total of 1,188 participants completed the survey, and 45% (*n* = 533) reported drinking alcohol. Thus, the present analysis included *N* = 533 individuals who reported drinking alcohol, 77% (*n* = 412) of whom reported using cannabis to treat a medical condition (CTT). Differences in sample characteristics between CTT and NCTT groups are described in Table 1.

### Alcohol Use Differences Between CTT and NCTT Groups

In all regression models below, slope values are reported as standardized regression coefficients (unstandardized betas are included in **Table 3**). Significance was set at *p* < 0.05. Controlling for gender, there was a significant association between the CTT vs. NCTT contrast *b* = 0.100, *t*_(521)_ = 2.266, *p* = 0.024 and drinking frequency. Examination of group means shows that the CTT group drank least often (Table 2). The CTT vs. NCTT contrast was not associated with any other outcome variables.

**Table 2:** Group Means for All Outcomes.

**Outcome of interest**	**CTT (*n* = 412), Mean (*SD*)**	**NCTT (*n* = 121), Mean (*SD*)**	
Drinking frequency	2.04 (1.9)	2.57 (2.1)	
Drinks per drinking occasion	2.89 (1.9)	3.31 (1.9)	
Co-use frequency	2.61 (1.5)	2.81 (1.5)	
Drink more or less on cannabis use days	1.73 (0.9)	1.90 (0.9)	
**Outcome of interest**	**Flower high-THC/CBD (*****n*** **=** **182), Mean (*****SD*****)**	**Flower medium-THC/CBD (*****n*** **=** **195), Mean (*****SD*****)**	**Flower low-THC/CBD (*****n*** **=** **113), Mean (*****SD*****)**
Drinking frequency	2.21 (2.1)	2.12 (1.8)	2.20 (2.1)
Drinks per drinking occasion	3.23 (2.1)	3.01 (1.8)	2.68 (1.7)
Co-use frequency	2.88 (1.6)	2.76 (1.3)	2.42 (1.6)
Drink more or less on cannabis use days	1.89 (0.9)	1.65 (0.9)	1.73 (0.9)
**Outcome of interest**	**Edible high-THC/CBD (*****n*** **=** **99), Mean (*****SD*****)**	**Edible medium-THC/CBD (*****n*** **=** **174), Mean (*****SD*****)**	**Edible low-THC/CBD (*****n*** **=** **143), Mean (*****SD*****)**
Drinking frequency	2.27 (2.0)	2.16 (2.0)	2.11 (2.0)
Drinks per drinking occasion	3.43 (1.8)	3.15 (2.0)	2.63 (1.8)
Co-use frequency	2.88 (1.4)	2.78 (1.5)	2.46 (1.6)
Drink more or less on cannabis use days	1.84 (0.9)	1.73 (0.9)	1.75 (0.9)

*Note that not every participant answered every question, so ns for each outcome may be less than total group ns listed*.

### Alcohol Use Differences Based on THC and CBD Content of Cannabis

Controlling for age and employment, Contrast 2 was associated with responses to the question “On the days when you use cannabis, do you usually drink more alcohol than usual, less alcohol than usual, or about the same amount?” *b* = 0.105, *t*_(475)_ = 2.329, *p* = 0.02. The High-THC/CBD group reported the highest scores (higher scores correspond to drinking more alcohol while lower scores indicate drinking less alcohol) and the medium-THC/CBD group reported the lowest scores^2^. In the model in which co-use frequency was the criterion, Contrast 1 was significant *b* = 0.121, *t*_(412)_ = 2.387, *p* = 0.017. Using flower-based groupings, neither contrast was associated with any other outcome variable.

Using the edible groupings, controlling for age, Contrast 1 was associated with DPDO *b* = 0.116, *t*_(406)_ = 2.360, *p* = 0.019 and co-use frequency *b* = 0.121, *t*_(357)_ = 2.220, *p* = 0.027. Using the edible-based grouping, neither contrast was significantly associated with any other outcome variable. All significant regression results are listed in Table 3.

**Table 3:** Results from regression models with significant group contrast effects.

**Model**	**Unstandardized B**	**Std Error**	**Standardized β**	***t***	***p***	***F***	***df***	***p***	***R*^**2**^**	**adj *R*^**2**^**
**Drinking frequency: CTT vs. NCTT**										
Overall model						3.722	2,521	**0.025**	0.014	0.010
Gender	0.208	0.176	0.052	1.178	0.239					
CTT vs. NCTT	0.240	0.106	0.100	2.266	**0.024**					
**Drink More/Less on Cannabis Days—Flower Groupings**										
Overall model						3.829	4,475	**0.004**	0.031	0.023
Age	0.006	0.003	0.095	2.002	**0.046**					
Employed	0.077	0.037	0.096	2.084	**0.038**					
Contrast 1	0.032	0.033	0.045	0.961	0.337					
Contrast 2	0.109	0.047	0.105	2.329	**0.020**					
**Frequency of alcohol** **+** **cannabis co-use—flower groupings**										
Overall model						1.502	4,412	0.201	0.014	0.005
Age	0.003	0.005	0.024	0.470	0.639					
Employed	0.002	0.068	0.001	0.028	0.978					
Contrast 1	0.141	0.059	0.121	2.387	**0.017**					
Contrast 2	0.057	0.085	0.033	0.665	0.506					
**Drinks per drinking occasion—edible groupings**										
Overall model						12.271	3,406	**<0.001**	0.083	0.076
Age	−0.033	0.007	−0.240	−4.947	**<0.001**					
Contrast 1	0.154	0.065	0.116	2.360	**0.019**					
Contrast 2	0.130	0.116	0.054	1.128	0.260					
**Frequency of alcohol** **+** **cannabis co-use—edible groupings**										
Overall model						1.652	3,357	**0.177**	0.014	0.005
Age	0.003	0.006	0.028	0.528	0.598					
Contrast 1	0.128	0.058	0.121	2.220	**0.027**					
Contrast 2	0.053	0.103	0.027	0.513	0.608					

*Bold font in p-value column indicates significant effects. In all models, Contrast 1 is the comparison of the Low-THC/CBD group to the other two groups, such that the Low-THC/CBD group is coded “−2,” and the Medium- and High-THC/CBD groups are both coded “1.” Contrast 2 is the comparison of the Medium- and High-THC/CBD groups, such that the Low-THC/CBD group is coded “0,” the Medium-THC/CBD group is coded “−1” and the High-THC/CBD group is coded “1”*.

## Discussion

Analyses demonstrated that CTT users drink less frequently than NCTT users, consistent with prior research demonstrating that medical cannabis use is associated with decreased drinking (5, 10, 11). No other differences emerged between these groups. It should be noted that categorization within the CTT group does not indicate strictly medical use. Being included in the NCTT group suggests recreational use, however, we did not explicitly ask about cannabis use motives. The lack of expected group differences may be due to the fact that these groups do not necessarily correspond to the medical and recreational groups tested in prior studies. Further, other factors not measured in this study (e.g., personality traits, social behaviors, lifestyle factors) may differ between these groups and contribute to this pattern of results.

We demonstrated that the THC/CBD ratio that participants consume in their typical flower and edible products impacts alcohol-related outcomes. Individuals who consume edibles containing lower THC/CBD ratios drink fewer DPDO and co-use less frequently compared to those using cannabis containing higher THC/CBD. Because individuals in the Low-THC/CBD group likely consumed a higher overall amount of CBD, this finding is consistent with preclinical literature suggesting that CBD reduces drinking and alcohol-seeking behavior (14–17). However, due to our retrospective design (and possible self-report bias and other limitations discussed in the limitations section), these data do not allow us to draw causal conclusions regarding the influence of THC or CBD on alcohol consumption.

Using the flower-based groupings, individuals in the High-THC/CBD group had higher scores on the question “*On the days when you use cannabis, do you usually drink more alcohol than usual, less alcohol than usual, or about the same amount?*” compared to the medium-THC/CBD group. Higher scores correspond to drinking more alcohol, and lower scores indicate drinking less alcohol on cannabis-using days. One explanation may be that it is not the THC/CBD ratio *per se* that impacts drinking more in a given sitting while using cannabis, but total THC or total CBD content. Future studies that could tightly control THC and CBD dose prior to an alcohol self-administration session could shed light on this relationship. Also note that in response to this question, all three cannabinoid groups reported drinking less alcohol on cannabis use days on average (see Table 2; note that a “1” response to this question corresponds to “much less alcohol” and a “2” corresponds to “a little less alcohol”), and no participant across the entire sample endorsed drinking “much more alcohol.” This suggests that cannabis users in this study are not at risk for drinking much more alcohol on the days that they use cannabis, regardless of the cannabinoid content of their typical products and whether or not they are using cannabis to treat a medical condition. Although intoxication was not explicitly measured in this study, cannabis may increase overall intoxication such that fewer drinks are needed for individuals to achieve their desired levels of intoxication. Consistent with this idea, one human alcohol and THC co-administration study found that THC combined with alcohol was associated with decreased participant ratings of wanting more alcohol, which suggests that cannabis may dampen or replace the desire to drink (34). Notably, individuals in the low-THC/CBD group co-used less frequently than those in the higher groups. This may be due to the less intoxicating properties of the lower-THC/CBD being less rewarding when combined with alcohol, although it could also reflect characteristics of the low-THC/CBD users, such as personality or lifestyle factors that impact the circumstances in which they use cannabis. Implications from these findings are limited, given that we did not assess the timespan during which individuals were using alcohol and cannabis each day. Future studies leveraging daily diary or Ecological Momentary Assessment methods could shed further light on the notion that cannabis intoxication may influence alcohol consumption.

## Limitations and Future Directions

This study has several methodological limitations. Data came from a convenience sample and relied on self-report. It is well-established that individuals tend to underreport substance use (35). The survey data is also subject to selection bias, as most individuals who participated were recruited through targeted social media ads as a result of “liking” cannabis-related content or through cannabis clinics. These participants were likely to be “pro-cannabis,” limiting our ability to generalize these results to individuals who have less experience with cannabis, who live in a state where cannabis has not been legalized, or who have a more neutral or negative attitude toward cannabis use. However, participant bias is a common limitation of online behavioral research and does not negate the utility of such data. Our sample was also limited in that it lacked racial diversity and was composed of 77% white individuals. This limits the extent to which results can be generalized to other populations. Future studies should include a more diverse population.

The survey did not ask about cannabis use motives (e.g., increasing social enjoyment, relaxation, stress-relief) beyond whether cannabis was used to treat a medical condition. This information would better characterize the sample and should be included in future studies. Further, there was scant prior data on which to base our classification of CTT and NCTT users. Individuals were classified as CTT users if they endorsed using cannabis to treat one or more major medical conditions for which medical cannabis is typically used (26, 27). These respondents may also use cannabis in situations in which they do not intend to treat a medical condition, as existing research suggests that recreational and medical motives for cannabis use often overlap. For example, over half of individuals using medical cannabis legally in the U.S. also report some recreational use (36). Thus, classification of cannabis users into distinct groups that accurately reflect their medical and recreational motives is a challenge across the field. Further research is needed to better understand how to make such classifications. The survey was also retrospective, and the accuracy of future studies could be improved through leveraging real-time data collection methods such as daily diaries or Ecological Momentary Assessment.

## Conclusions

Results suggest that using cannabis to treat a medical condition, and the THC/CBD content of flower and edible cannabis people use, play a role in determining the relationship between cannabis use and alcohol consumption. Future studies are needed to better understand this association. In particular, future research would ideally include participants that fall into more clearly defined and distinct medical and recreational groups. Research that involves daily assessments to better understand the temporal associations between alcohol and cannabis use, and laboratory studies in which alcohol is co-administered alongside tightly-controlled THC and CBD doses will be necessary to draw meaningful conclusions about the nature of these relationships.

## Data Availability Statement

The raw data supporting the conclusions of this article will be made available by the authors, without undue reservation.

## Ethics Statement

The studies involving human participants were reviewed and approved by University of Colorado Institutional Review Board. The patients/participants provided their written informed consent to participate in this study.

## Author Contributions

RM and KH developed and implemented the online survey. RM and CA prepared data and created relevant study variables. CA and HK conducted data analysis. HK conceived of the study idea and wrote the manuscript. All authors contributed to the article and approved the submitted version.

## Conflict of Interest

The authors declare that the research was conducted in the absence of any commercial or financial relationships that could be construed as a potential conflict of interest.

## Footnotes

^1^Note that for all outcome variables, response options were ordinal, Likert-style scales. However, because all questions included 5 or more ordered response options, these variables were treated as continuous data (30–33) and thus were appropriate dependent variables for the OLS regression approach. For alcohol frequency, 11 response options ranged from less than once a month to daily, which corresponded to values of 1–7. For DPDO, 10 response options ranged from 1 drink to “10 or more drinks” coded as 1–10. For “*On the days when you use cannabis, do you usually drink more alcohol than usual, less alcohol than usual or about the same amount*,” there were 5 response options ranging from “much less alcohol” to “much more alcohol” and coded from 1 to 5. For alcohol and cannabis co-use frequency, there were 7 response options ranging from “Never” to “Everyday” and coded from 0 to 6.

^2^Specifically, in the Low-THC/CBD group, 54.9% reported drinking much less, 20.4% reported drinking a little less, 22.1% reported drinking about the same amount, 2.7% reported drinking a little more, and 0% percent reported drinking much more. In the Medium-THC/CBD group, 58.5% reported drinking much less, 17.4% reported drinking a little less, 21.0% reported drinking about the same amount, 1.5% reported drinking a little more, 0% percent reported drinking much more and 1.5% did not answer. In the High-THC/CBD group, 47.8% reported drinking much less, 15.4% reported drinking a little less, 34.6% reported drinking about the same amount, 1.1% reported drinking a little more, 0% percent reported drinking much more, and 1.1% did not to answer.
